# The Effects of Adipose Tissue Dysregulation on Type 2 Diabetes Mellitus

**DOI:** 10.3390/biomedicines13071770

**Published:** 2025-07-19

**Authors:** Jamie Rausch, Kaitlyn E. Horne, Luis Marquez

**Affiliations:** 1 School of Nursing, Indiana University, Fort Wayne Campus, Fort Wayne, IN 46805, USA; 2 Independent Researcher, Fort Wayne, IN 46805, USA

**Keywords:** adipose tissue dysregulation, type 2 diabetes mellitus, leptin, adiponectin, leptin-to-adiponectin ratio, insulin resistance, insulin sensitivity, chronic inflammation, chronic disease

## Abstract

Internationally, the prevalence of type 2 diabetes mellitus (T2DM) and obesity rates are increasing significantly. As these epidemics continue to spread, the continuation of further research is paramount given that chronic diseases, such as T2DM, cause strain on both economies and healthcare systems. Recently, adipose tissue has been identified as an endocrine organ that produces many hormones that influence many bodily processes. Adipose tissue dysregulation (ATD)—when adipokines (adipose tissue hormones) are produced in abnormal amounts—plays an important role in T2DM development, progression, and prognosis. This narrative review focuses on mechanisms linking ATD with T2DM through adipokine actions (specifically, leptin and adiponectin) on insulin resistance and glucose metabolism. Here we show that the adipokines leptin and adiponectin are valuable in monitoring, diagnosing, and treating diseases. Further, their ratio (the leptin-to-adiponectin ratio, or LAR) may be more valuable than either adipokine individually. The LAR may give researchers the ability to utilize a primary prevention approach by utilizing LAR as a biomarker influencing early prognosis and treatment. Targeting ATD through diet, weight loss, physical activity, etc., may improve prevention and management outcomes for patients living with or at risk of T2DM.

## 1. Introduction

For nearly four decades, there has been an understanding that excess adipose tissue is positively, directly correlated with a diagnosis of type 2 diabetes mellitus (T2DM) [1,2,3,4]. Despite knowing this, the exact mechanism through which adipose tissue dysregulation (ATD) stimulates diabetes disease formation and progression has not been fully elucidated. With the discovery of adipose tissue functioning as an endocrine organ [5,6,7,8], further details of the pathway have been identified.

The relationship between hormones (e.g., insulin, leptin, and adiponectin) and dietary factors (e.g., glycemic load and energy intake) influences the development and progression of T2DM [9,10,11,12,13]. The main function of insulin is to lower blood glucose by promoting glucose uptake in cells [9,10,11]. Unfortunately, a significant indication of T2DM is the presence of insulin resistance when cells no longer effectively respond to insulin signaling, which results in hyperglycemia [9,10,11,12]. Leptin, produced mainly by adipocytes, suppresses appetite and increases energy expenditure, leading to regulated energy balance [14,15,16]. Obesity and overnutrition elevate leptin levels but lead to leptin resistance, which dulls the regulatory effects of leptin, further contributing to weight gain and insulin resistance [15,16]. Dietary regulatory actions, such as energy intake and glycemic load, further complicate the situation. The consumption of a high-calorie diet increases leptin secretion and contributes to leptin resistance [15,16,17]. The glycemic load indicates the effect of total carbohydrate intake on glucose levels [18]. High-glycemic-load diets generate spikes in blood glucose and insulin, which can modify leptin signaling, creating a metabolic imbalance [18]. Meanwhile, adiponectin improves insulin sensitivity and wields anti-inflammatory actions [19,20,21]. The inverse of the effect on leptin, increased adiposity results in decreased adiponectin levels [22,23]. Further, decreased adiponectin levels are concomitant with increased insulin resistance and an increased risk of T2DM [19,20,21]. The interconnected hormonal and dietary factors augment metabolic dysfunction, facilitating the diagnosis and progression of T2DM. (Table 1 lists categories and factors that are related to both T2DM and adipose tissue.)

The purpose of this paper is to review the current literature related to T2DM prevalence, the influence of T2DM on public health, ATD and how it effects T2DM, and why this connection is important to consider in future research and clinical practice.

## 2. Materials and Methods

**Figure 1:**
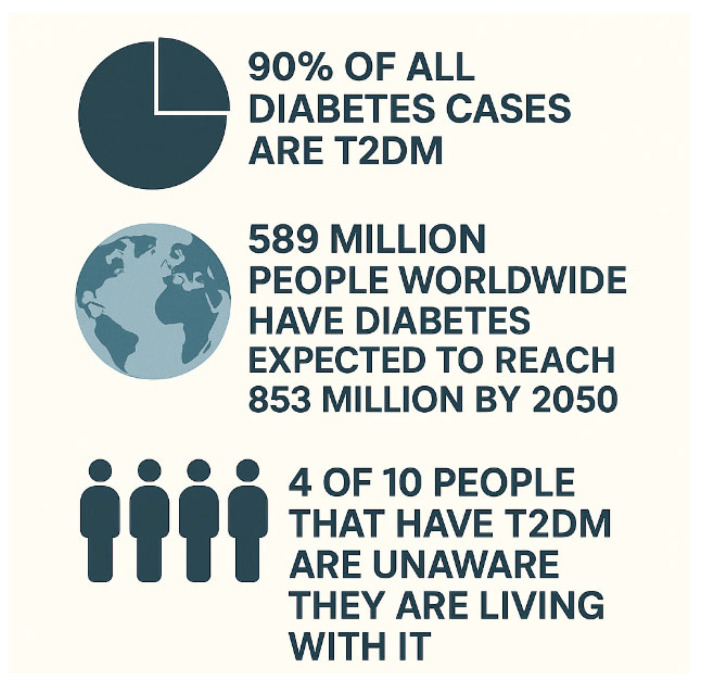
Type 2 diabetes mellitus (T2DM) facts.

**Figure 2:**
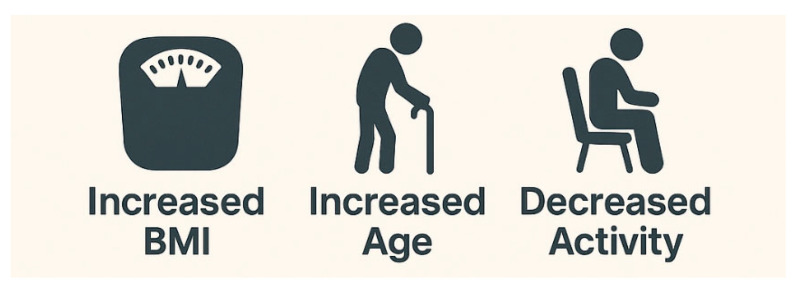
Key contributors to the rise in type 2 diabetes mellitus (T2DM) include (but are not limited to) the above.

This narrative review was aided by using ChatGPT (OpenAI, San Francisco, CA, USA, https://openai.com/chatgpt/overview/, accessed on 28 May 2025) to create outlines to guide the paper and in the creation of Figure 1 and Figure 2 on 8 July 2025. The articles used in the review were selected from Google Scholar and PubMed by using the following terms in our search: leptin, adiponectin, inflammation, aging, type 2 diabetes mellitus, adipose tissue dysregulation, insulin, and insulin sensitivity.

Figure 1 and Figure 2 note: The data presented in these graphics were obtained from the International Diabetes Federation (IDF) [48]. This graphic was made with the assistance of ChatGPT (OpenAI, San Francisco, CA, USA) using data from the IDF on 8 July 2025.

## 3. Overview of T2DM

### 3.1. Disease Description and Pathophysiology of T2DM

Discussing T2DM pathophysiology is a critical component in understanding and mitigating ATD. In line with the latter, it is important to note that type 1 diabetes mellitus (T1DM) accounts for only about 5 to 10% of newly diagnosed cases of diabetes mellitus [49], whereas T2DM can be attributed to 90 to 95% of newly diagnosed cases of diabetes [49]. A chronic metabolic disorder resulting from multiple pathophysiological pathways, T2DM is indicated by defects in insulin secretion and uptake, regularly elevated levels of blood glucose levels, or both [50,51,52,53,54]. Diagnosis occurs when an individual has one or more of the following indicators: glycated hemoglobin (HbA1c) ≥ 6.5%, fasting blood glucose ≥ 126 mg/dL, or 2 h, post-prandial glucose ≥ 200 mg/dL [54]. T2DM often occurs as a comorbid condition in individuals with other chronic diseases such as obesity, cardiovascular disease, and depression [55,56,57]. Recently, T2DM has seen an unexpected rise in both adolescents and children, in part due to increased rates of global obesity in all age groups [47,58,59].

T2DM is highly influenced by the blending of genetic, lifestyle, and environmental risk factors that contribute to inflammation and ATD. Genetics, passed from generation to generation, predispose inheriting individuals to obesity, insulin resistance, and even inflammation [60]. Lifestyle choices, such as consuming the inflammatory Western diet, little to no physical activity, and excessive stress, can intensify these complications [46]. Inflammation is further aggravated by environmental factors (e.g., pollution) and contribute to T2DM development [61,62]. The convergence of these factors directly lead to insulin resistance, inflammation, and T2DM.

### 3.2. Prevalence and Influence on Public Health

Data extracted from the Global Burden of Disease database indicates that the global burden of T2DM was steadily increasing from 1990 to 2019 [63]. Internationally, the prevalence rate of T2DM in 2021 was 10.5% [64]. It is expected to grow to 11.3% by 2030 and, subsequently, 12.2% by 2040 [64]. Thus, without significant public health interventions, these rates are likely to continue to increase significantly for the foreseeable future. (Figure 1 and Figure 2 below provide more information about T2DM).

When age-adjusted, the incidence rates for T2DM have been positively correlated with the Sociodemographic Index (SDI) [63], “a summary measure that identifies where countries or other geographic areas sit on the spectrum of development” [65]. Much of this correlation can be attributed to how rapid and significant the increase is that has occurred globally over the last several decades. To keep up with high levels of demand and extend shelf life, many food products have become highly processed [66]. Thus, excessive amounts of fats and sugars used in production are being consumed daily by most populations [66]. This rapid urbanization has also adjusted peoples’ lifestyles through changes to their physical environment that result in increased air pollution and decreased physical activity [67,68].

Each of these lifestyle factors place significant stress on regional healthcare systems, especially those in low-SDI regions. Though this is a problem on a global scale, it is disproportionately exaggerated in low-SDI communities with decreased access to all forms of healthcare. These communities exhibit decreased compliance to provided management regimens (sometimes related to the ability to purchase needed supplies or medications), directly correlating to an increased burden of disease [63].

Surprisingly, low-SDI regions had a lower incidence of T2DM compared to high-SDI regions [63]. When taken at face value, this may be surprising; however, it should be considered that high-SDI regions have more access to detection methods, leading to earlier diagnoses, management, and, subsequently, better outcomes [63]. It should be noted that there are conflicting data regarding the relationship between SDI and prevalence; while some exhibit no relationship, others indicate a positive relationship between growing socioeconomic development and prevalence [63,69].

## 4. Role of Adipose Tissue in Metabolic Regulation

### 4.1. Functions of Adipose Tissue

#### 4.1.1. Normal Adipose Tissue Function

The initial perception of adipose tissue function was that it served as an inert storage of energy in the form of triacylglycerol, or triglycerides, the primary form of dietary lipids from fats and oils [70]. Simply put, adipose tissue stored excess fat, released stored fat when dietary intake decreased to meet physical demands, assisted in temperature regulation, and protected internal organs [70].

The secretory role of adipose tissue is a newer realization. In the 1980s the role of adipose tissue as an endocrine organ was identified [70,71,72]. Adipose tissue secretes adipokines, hormones released from adipose tissue that go on to influence other bodily actions, such as metabolism and inflammation [73]. Two of these hormones, leptin and adiponectin, have been identified as important regulators of inflammation [42]. In normal-weight individuals, leptin and adiponectin are released in regulated levels that maintain homeostasis [74]. Despite this knowledge, normal levels of leptin and adiponectin exist within very limited parameters (see Table 2). The leptin-to-adiponectin ratio (LAR) is so novel that normal levels have yet to be determined.

#### 4.1.2. Dysregulation Mechanisms

ATD happens very easily, often without any indication of change. As an individual gains excess weight, as seen in obesity and aging, cells of adipose tissue (adipocytes) become larger (hypertrophy) and more numerous (hyperplasia) [42]. These enlarged adipocytes produce irregular amounts of leptin and adiponectin. Leptin, which has mostly pro-inflammatory actions, is produced in greater amounts than normal [77]. In contrast, adiponectin, which has mostly anti-inflammatory actions, is produced in lesser amounts than normal [78]. The changes in the levels of leptin and adiponectin lead to systemic inflammation that contributes to disease development and progression in T2DM.

#### 4.1.3. Preventing ATD

Preventing ATD is imperative to reducing metabolic disease risk, including T2DM [79,80]. Common prevention strategies include nutrition and dietary interventions; physical activity; weight management; calorie restriction; improved sleep; and stress reduction. Plant-based or Mediterranean diets and supplements (e.g., fish oil) support normal adipocyte function and attenuate pro-inflammatory adipokine and cytokine production [81,82,83]. Increasing physical activity can decrease ATD and improve hormone levels [84,85,86,87]. Adipocyte hypertrophy and hyperplasia can be prevented by retaining a healthy weight and managing caloric intake [88,89]. Improved sleep hygiene and stress reduction activities encourage healthy function of adipose tissue [90,91,92,93]. Combining these elements promote balance to metabolic and inflammatory actions of adipose tissue improving risk of disease development.

Furthermore, it is important to consider that ATD can be reversed. Recent studies indicate that weight loss in overweight or obesity decreases the effects of ATD. In fact, Moreira and colleagues determined that following Roux-en-Y gastric bypass surgery, weight loss was associated with significantly increased adiponectin levels and significantly decreased leptin and glucose levels [94]. Another study showed similar significance in leptin and adiponectin levels, while some markers of inflammation showed no significant changes (e.g., IL-10, IL-1β, IL-6, TNF-α) [89]. Additionally, two systematic reviews and meta-analyses on exercise showed conflicting results. The results of the 2021 study by Jadhav and colleagues found that physical activity reduced leptin levels but had no significant change in adiponectin levels [95]. In contrast, the results of the 2023 study by Garcia-Hermoso and colleagues revealed that physical exercise significantly improved both leptin levels (decreased) and adiponectin levels (increased), which further improved glucose levels [96]. These studies indicate that physical exercise should be encouraged in persons with obesity or T2DM to improve ATD and glucose levels.

## 5. Inflammatory Responses and Insulin Signaling Pathways

The process of systemic inflammation as it relates to adiposity and insulin resistance begins with the hypertrophy and hyperplasia of adipocytes, specifically within visceral fat. Increased visceral fat, which envelopes vital organs, releases pro-inflammatory adipokines like tumor necrosis factor (TNF)-α and interleukin (IL)-6, which, in turn, lower levels of adiponectin by downregulation [37,38,39,40,41]. As adipocytes expand and proliferate, the surrounding blood vessels cannot match pace with their growth, thus leading to adipocyte hypoxia [40]. Monocytes circulating in the bloodstream sense the dead or dying adipocytes via chemokines [40]. Macrophages from the bloodstream join adipose tissue macrophages (ATMs), or resident macrophages, to surround and phagocytize necrotic adipocytes in a crownlike structure [38]. It is known that, in healthy adipose tissue, the proportion of ATMs is only about 5–10%, whereas in obese individuals. this number may be as high as 50% [41,97]. It has been shown that TNF-α and IL-6 compared against adiponectin exhibit a negative correlation, contributing to T2DM formation [98]. The continued cycle of hypertrophy and hyperplasia perpetuates the state of low-grade chronic inflammation via inflammatory cytokines.

Chronic inflammation in adipose tissue, caused by ATD, plays a significant role in insulin resistance development, which is a hallmark of metabolic disorders, including T2DM. The mechanisms linking adipose tissue inflammation to insulin resistance consist of the infiltration of immune cells, pro-inflammatory cytokine release, and signaling pathway disturbance [24]. Adipose tissue expands past its corporal capacity during obesity, leading to cellular stress, oxidative stress, and hypoxia [99]. The resulting pro-inflammatory environment is portrayed by a shift in macrophage polarization (from an anti-inflammatory M2 phenotype to a pro-inflammatory M1 phenotype), reinforcing the chronic inflammatory state [100]. Furthermore, the infiltration and activation of T cells and B cells are increased, amplifying cytokine production and disrupting insulin signaling [100].

## 6. Clinical Implications of ATD in T2DM

Dysregulated adipose tissue exacerbates T2DM symptoms and complications in a variety of ways. First, as the pro-inflammatory cytokines (e.g., TNF-a and IL-6) are secreted by ATD, they interfere with the signaling of insulin receptors, which results in insulin resistance [36]. As previously stated, ATD sustains a pro-inflammatory environment with changes to macrophage polarization and increased T and B cell activity, further perpetuating insulin resistance [100]. Glucose metabolism and insulin sensitivity are disrupted by decreased adiponectin production and increased leptin production [101]. Lipotoxicity (the accumulation of transitional lipid materials in non-adipose tissues) throughout liver, muscles, and pancreas results in altered insulin signaling and pancreatic β-cell function [25]. Insulin resistance in muscle and fat tissue diminishes glucose uptake at a cellular level, leading to persistent hyperglycemia [102]. Further, pancreatic β-cells damaged from excessive inflammatory mediators and lipotoxic stress from ATD reduce the production of insulin over time [103].

**Figure 3:**
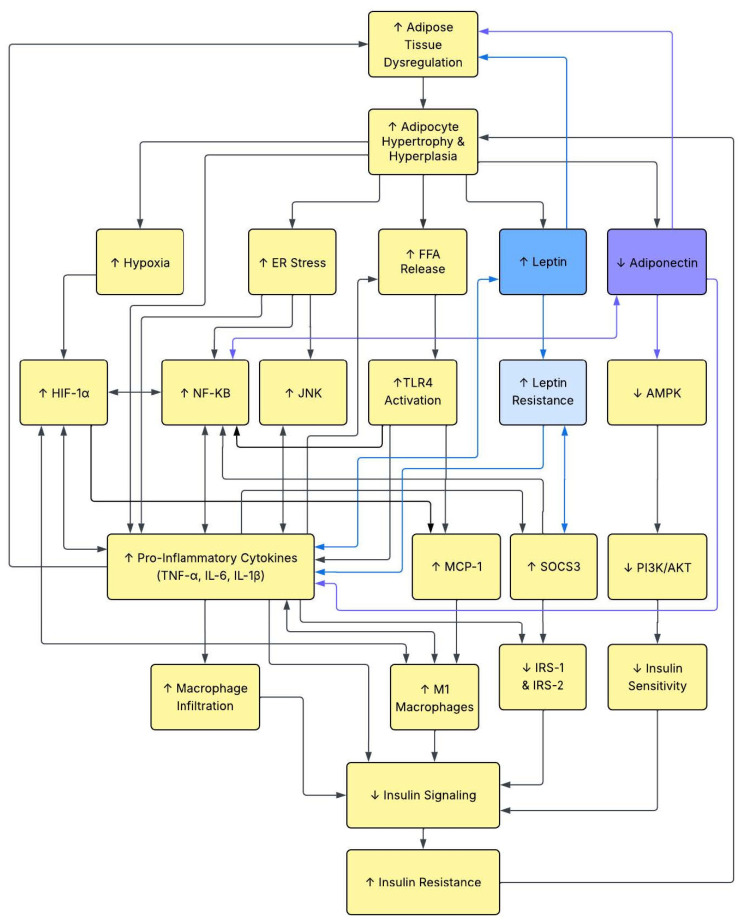
Pathophysiological pathways from adipose tissue dysregulation (ATD) to insulin resistance. Note: The complete mechanistic pathway between adipose tissue disfunction (ATD) and insulin resistance is beyond the scope of this paper; however, a brief summary is warranted. ATD begins with adipocyte hypertrophy and hyperplasia [26,27,28,29] in visceral fat and continues through several distinct pathways. Highlights of the pathways include the following: (1) increased hypoxia: larger adipocytes outgrow the oxygen supply leading to hypoxia [26,28,30]; (2) increased endoplasmic reticulum (ER) stress, resulting in accumulated proteins, known as the unfolded protein response [28,30,31,107]; (3) Increased free fatty acid (FFA) release, leading to changes in metabolism and inflammation [27,30,31,107,108]; (4) increased leptin levels [26,27,28,30,31,32,33,34,35]; and (5) decreased adiponectin levels [26,28,30,31,32,35]. These pathways continue to alter normal functioning by causing increased levels of the following: hypoxia-inducible Factor (HIF)-1a [28,30,31,33]; action of the nuclear factor kappa-light-chain-enhancer of activated B cells (NF-KB) [26,29,30,31,33,107] and c-Jun N-terminal kinase (JNK) pathways [30,31,33,35,107,108]; toll-like receptor (TLR)4 activation [29,30,31,33,107]; leptin resistance [26,27,34,35,109,110]; pro-inflammatory cytokine (e.g., TNF-α, IL-6, IL-1β) production [26,30,31,32,33,107,111]; monocyte chemoattractant protein (MCP)-1 [30,31,33,108] and suppressor of cytokine signaling (SOCS)3 actions [26,31,34]; macrophage infiltration into adipose tissue [26,27,28,30,31,32,33,35]; and conversion of macrophages to an M1 profile [26,27,28,30,31,32,33,35]. In addition, they cause decreased levels of the following: AMP-activated protein kinase (AMPK) [26,27,30,31,107,108,112] and phosphoinositide 3-kinese and protein kinase B (PI3K/AKT) pathway signaling [26,27,28,29,30,31,33,35,107,108,111]; insulin receptor substrate (IRS) 1 and 2 actions [27,30,31,107,108]; insulin sensitivity [27,31,107,108]; and insulin signaling [27,31,107,108]. These alterations result in insulin resistance [26,28,29,30,31,32,33,107,108,111], a hallmark of type 2 diabetes mellitus (T2DM) [26,30,31,32,111]. Furthermore, as seen in the diagram, many of the actions have feedback loops that further exacerbate dysregulation and disease processes. Please note that this is not an exhaustive diagram of all mechanistic actions. Single direction arrows indicate a one-directional pathway, while double arrows signify when feedback loops are involved. Yellow colored blocks indicate steps in the pathway involved between ATD and T2DM but not the focus of this paper. Leptin and actions immediately involved with it are indicated by blue coloring in blocks and arrows. Adiponectin and actions immediately involved with it are indicated by purple coloring in blocks and arrows.

Additionally, ATD results in the promotion of comorbidities which add to the inflammation exhibited throughout the body. ATD contributes to vascular endothelial damage through inflammatory mediators, exacerbating cardiovascular disease risk [104]. Increased risk of heart attack and stroke result from ATD’s increased dyslipidemia and inflammation which accelerates arterial plaque buildup [105]. ATD also adds to the development and progression of hypertension, dyslipidemia, and non-alcoholic fatty liver disease [106]. Figure 3, below, shows a more in-depth molecular-level schematic of the pathway from ATD to insulin resistance.

## 7. Leptin in T2DM

### 7.1. Leptin Physiology—Normal Function

Leptin, produced and secreted primarily by adipocytes, contributes significantly to the regulation of energy balance, hunger, and metabolism by signaling the hypothalamus in the brain to suppress appetite and increase energy expenditure [113]. Typically, in lean individuals, leptin enhances insulin sensitivity (in tissues such as liver and muscle) and promotes glucose uptake and fatty acid oxidation, reducing insulin resistance [36,113,114]. Energy homeostasis and metabolism result from symbiotic effects of leptin and insulin [115,116]. Leptin directly induces pancreatic β-cells and constrains insulin secretion in certain conditions and may protect against oxidative stress and lipotoxicity in β-cells [113,114].

### 7.2. Leptin Pathophysiology—Dysregulation

Leptin resistance is often present in obesity and T2DM. Circulating leptin levels are higher than normal in leptin resistance; however, leptin sensitivity at its receptors is reduced [113]. The brain then fails to respond to the appetite-suppressing effects of leptin and the result is hyperphagia (overeating), weight gain, and exacerbated insulin resistance [113,117]. As such, leptin resistance leads to a perverse cycle that intensifies metabolic dysfunction. Further heightening hyperglycemia, dysregulation of the pancreatic β-cell signaling pathway by leptin may impair insulin production in T2DM [117]. In obesity and T2DM, leptin also acts as a pro-inflammatory cytokine, causing an inflammatory ripple effect throughout the body [37,113,114,117].

### 7.3. Therapeutic Implications - Leptin

Despite the fact that normal leptin affects appetite and metabolism, leptin resistance creates a barrier to using leptin alone as a therapeutic agent in T2DM.

## 8. Adiponectin in T2DM

### 8.1. Adiponectin Physiology—Normal Function

Adiponectin, an adipokine (hormone) secreted mostly by subcutaneous adipose tissue, provides vital functions in the regulation of glucose and lipid metabolism and insulin sensitivity in normal circumstances [37,114,118]. Insulin sensitivity is enhanced by adiponectin in several ways. First, adiponectin improves the skeletal muscle uptake of circulating glucose [37,114]. Next, adiponectin activates the AMPK (AMP-activated protein kinase) pathway to increase fatty acid oxidation [37,114]. Finally, adiponectin acts directly to suppress glucose production by the liver [37]. Further, adiponectin engages in known anti-inflammatory and anti-atherogenic actions. For instance, adiponectin reduces the production of pro-inflammatory cytokines, such as tumor necrosis factor (TNF)-α [114], and stimulates the production of anti-inflammatory cytokines, such as interleukin (IL)-10 [37,114]. Moreover, oxidative stress and endothelial dysfunction are reduced by adiponectin [37,114].

### 8.2. Adiponectin Pathophysiology—Dysregulation

In obesity, insulin resistance, and T2DM, adiponectin levels are significantly decreased, resulting in reduced insulin sensitivity, glucose uptake, and fatty acid metabolism [37,114]. Reductions in adiponectin levels further result in increased glucose output from the liver, worsening hyperglycemia [114,119]. Low levels of adiponectin relate to chronic low-grade inflammation, endothelial dysfunction, and increased risk of cardiovascular diseases [119]. Although adiponectin, like leptin, is produced by adipocytes, adiponectin is inversely correlated with the amount of body fat [119]. Additionally, target tissues may exhibit reduced responsiveness to normal adiponectin levels in adiponectin resistance [119]. In some cases, adiponectin has shown some pro-inflammatory actions in what is referred to as the adiponectin paradox [114,118,119,120].

### 8.3. Therapeutic Implications - Adiponectin

Due to its mostly anti-inflammatory effects, adiponectin offers an opportunity to improve health and disease risk, especially in T2DM, by targeting it with therapeutic interventions. Several lifestyle modifications offer natural increases to adiponectin levels. Increasing the dietary intake of omega-3 fatty acids elevates adiponectin levels [121], as do exercise and weight loss [119]. Pharmaceuticals have also shown promise in improving circulating adiponectin levels. For example, thiazolidinediones (TZDs), sodium-glucose cotransporter-2s (SGLT2)s, gastric inhibitory polypeptides (GIPs), glucagon-like peptide-1s (GLP-1s), angiotensin-converting enzyme (ACE) inhibitors, angiotensin receptor blockers (ARBs), statins, and fibrates increase adiponectin levels [119]. Furthermore, new investigational therapies, like adiponectin receptor agonists (AdipoRons), gene therapy, and peptide analogs, target adiponectin pathways for therapeutic benefits [119,122]. Adiponectin monitoring may help predict risk, track therapeutic response, and even predict disease progression [114,119,123].

## 9. Leptin-to-Adiponectin Ratio (LAR) in T2DM

### 9.1. Physiology—Normal Function

The leptin-to-adiponectin ratio, or LAR, is a composite biomarker that represents the balance between leptin (pro-inflammatory, insulin-resistance-promoting) and adiponectin (anti-inflammatory, insulin-sensitizing) [119,121,124]. In healthy individuals, normally, low leptin levels and high adiponectin levels result in a low LAR and are reflective of metabolic homeostasis, normal insulin sensitivity, and low inflammation [124,125]. As such, the LAR provides an integrated view of total adipose tissue function compared to the use of leptin or adiponectin alone.

### 9.2. Pathophysiology—Dysregulation

As previously discussed, with obesity and T2DM, leptin levels increase and adiponectin levels decrease, leading to higher LARs. Higher LARs indicate ATD and increased insulin resistance, worsened glycemic control, endothelial dysfunction, a heightened pro-inflammatory state, and a risk of cardiovascular diseases [124,125].

### 9.3. Therapeutic Implications

Due to its more accurate indication of ATD, the LAR presents a strong opportunity for a clinical biomarker in predicting risk for insulin resistance and T2DM. Additionally, the LAR could provide feedback in treatment monitoring for lifestyle and pharmacological interventions. In using the LAR as a biomarker, goals should include reducing leptin levels and increasing adiponectin levels. Interventions that have shown improvement in the LAR include weight loss, exercise, dietary modifications, bariatric surgery, and medications [94,120,126]. Furthermore, the LAR could be used to identify those individuals at high risk prior to disease onset. When interventions are implemented to improve the LAR, it is possible to prevent tissue and organ damage before it occurs. (Table 3 provides a summary of leptin, adiponectin, and the LAR in the context of T2DM.)

## 10. Conclusions

In sum, ATD plays a significant role in T2DM. Alterations to hormones, chronic inflammation, and increased pathological fat distribution disturb insulin sensitivity and glucose metabolism, which further emphasizes that metabolic dysfunction is a hallmark of T2DM. Early interventions targeting nutrition, physical activity, and weight management maintain healthy adipose tissue function and may delay, or even prevent, T2DM. Future research should explore the prevention and reversal of ATD, evaluating long-term effects on T2DM prevention. Further, leptin, adiponectin, and the LAR offer opportunities for preventative biomarkers of ATD and T2DM. Addressing ATD may be a crucial step in improving health outcomes and transforming the prevention and management of T2DM worldwide.

## Figures and Tables

**Table 1:** Factors associated with type 2 diabetes mellitus (T2DM) and adipose tissue.

Category	Factor	Relationship to T2DM	Connection to Adipose Tissue
Physiological	Insulin resistance [24]	Central mechanism to T2DM development	Increased visceral fat impairs insulin signaling
	β-cell dysfunction [25]	Leads to reduced insulin secretion	Inflammation from adipose tissue can damage β-cells
Hormonal	Leptin [26,27,28,29,30,31,32,33,34,35]	Often elevated in obesity but with resistance	Secreted by adipocytes; dysregulation linked to insulin resistance
	Adiponectin [26,28,30,31,32,35]	Decreased levels associated with T2DM	Produced by adipose tissue; enhances insulin sensitivity
Inflammatory	TNF-α, IL-6 [36]	Increased inflammation and insulin resistance	Secreted by adipose tissue macrophages during obesity
Morphological	Visceral adiposity [37,38,39,40,41]	Strong predictor of metabolic dysfunction	Excess intra-abdominal fat is metabolically active and pro-inflammatory
	Adipocyte hypertrophy [42]	Enlarged fat cells impair glucose metabolism	Associated with increased inflammatory cytokine secretion
	Ectopic fat deposition [43,44,45]	Fat in liver, muscle, or pancreas impairs function	Occurs when adipose tissue is unable to store excess energy
Lifestyle and Environmental	High-calorie diet [46]	Promotes weight gain and insulin resistance	Increases adipose tissue mass and dysregulation
	Physical inactivity [46]	Reduces glucose uptake and promotes insulin resistance	Limits adipose tissue lipolysis and increases fat storage
Genetic	Family history of T2DM [47]	Increases individual risk	Some gene variants affect adipose tissue development and function

Note: above is not an exhaustive list of the potential relationships between T2DM and adipose tissue.

**Table 2:** Normal values for leptin and adiponectin levels.

Sex/BMI	Leptin (ng/mL)	Adiponectin (mcg/mL)
=22	0.5–12.5	
<25		5–37
25–30		5–28
>30		2–20
=22	0.5–15.2	
<25		5–37
25–30		4–20
>30		4–22

Note: The normal values listed in the table above were available from the Cleveland Clinic [75,76]. All other values for leptin and adiponectin are currently unknown. There are no currently known reference values for the leptin-to-adiponectin ratio (LAR).

**Table 3:** Summary table of leptin, adiponectin, and the leptin-to-adiponectin ratio (LAR) as they pertain to type 2 diabetes mellitus (T2DM).

Aspect	Leptin [26,27,28,30,31,32,33,34,35]	Adiponectin [26,28,30,31,32,35]	LAR [94,119,120,121,124,125,126]
Source	Primarily adipose tissue	Primarily adipose tissue	Derived ratio (leptin/adiponectin)
Normal Role	Regulates appetite and energy expenditure, and enhances insulin sensitivity	Enhances insulin sensitivity, is anti-inflammatory, and promotes lipid oxidation	Reflects balance between pro- and anti-diabetic/inflammatory adipokines
Levels in T2DM	*Increased*—due to adiposity and leptin resistance	*Decreased*—due to increased adiposity	*Increased*
Effect on Insulin	*Decreases*—when resistance develops	*Increases*	High LAR correlates with *greater insulin resistance*
Inflammatory Role	*Pro-inflammatory*	*Anti-inflammatory*	High LAR = *Pro-inflammatory state*
Clinical Relevance	Marker of adiposity, leptin resistance, and inflammation	Marker of insulin sensitivity, metabolic health, and inflammation	*Better predictor* of T2DM risk than either alone
Therapeutic Targeting	Indirect: weight loss, others possible but not currently clear	Targeted by changes to diet, exercise, and pharmacological interventions	Lowered through lifestyle changes, insulin-sensitizing therapy, and modifications of leptin and/or adiponectin
Predictive Value	Moderate alone	Moderate alone	*High predictive value* for T2DM

Note: LAR = leptin-to-adiponectin ratio; T2DM = type 2 diabetes mellitus.

## Abbreviations

The following abbreviations are used in this manuscript (in order of appearance):

T2DM	Type 2 Diabetes Mellitus
ATD	Adipose Tissue Dysregulation
T1DM	Type 1 Diabetes Mellitus
HbA1c	Glycated Hemoglobin
IDF	International Diabetes Federation
SDI	Sociodemographic Index
LAR	Leptin-to-Adiponectin Ratio
BMI	Body Mass Index
TNF	Tumor Necrosis Factor
IL	Interleukin
ATMs	Adipose Tissue Macrophages
ER	Endoplasmic Reticulum
FFA	Free Fatty Acids
HIF	Hypoxia-Inducible Factor
NF-KB	Nuclear Factor Kappa-light-chain-enhancer of activated B cell
JNK	c-Jun N-terminal Kinase
TLR	Toll-Like Receptor
SOCS	Suppressor Of Cytokine Signaling
AMPK	AMP-activated protein Kinase
PI3K/AKT	Phosphoinositide 3-kinese and protein kinase B
IRS	Insulin Receptor Substrate
TZDs	Thiazolidinediones
SGLT2	Sodium-Glucose cotransporter-2s
GIPs	Gastric Inhibitory Polypeptides
GLP-1s	Glucagon-like Peptide 1s
ACE	Angiotensin-Converting Enzyme
ARB	Angiotensin Receptor Blocker
